# Author Correction: Curved neuromorphic image sensor array using a MoS_2_-organic heterostructure inspired by the human visual recognition system

**DOI:** 10.1038/s41467-022-32731-0

**Published:** 2022-09-01

**Authors:** Changsoon Choi, Juyoung Leem, Minsung Kim, Amir Taqieddin, Chullhee Cho, Kyoung Won Cho, Gil Ju Lee, Hyojin Seung, Hyung Jong Bae, Young Min Song, Taeghwan Hyeon, Narayana R. Aluru, SungWoo Nam, Dae-Hyeong Kim

**Affiliations:** 1 Center for Nanoparticle Research, Institute for Basic Science (IBS), Seoul, 08826 Republic of Korea; 2 School of Chemical and Biological Engineering, Institute of Chemical Processes, Seoul National University, Seoul, 08826 Republic of Korea; 3 Department of Mechanical Science and Engineering, University of Illinois at Urbana-Champaign, Urbana, IL 61801 USA; 4 School of Electrical Engineering and Computer Science, Gwangju Institute of Science and Technology, Gwangju, 61005 Republic of Korea; 5 Department of Materials Science and Engineering, University of Illinois at Urbana-Champaign, Urbana, IL 61801 USA; 6 Department of Materials Science and Engineering, Seoul National University, Seoul, 08826 Republic of Korea

**Keywords:** Electronic devices, Sensors and biosensors, Two-dimensional materials

Correction to: *Nature Communications* 10.1038/s41467-020-19806-6, published online 23 November 2020

Since the publication of this work, Minsung Kim has changed their name from Min Sung Kim. This has now been amended.

