# Supplementary material for: Curved neuromorphic image sensor array using a MoS2-organic heterostructure inspired by the human visual recognition system
Source: Nat Commun. 2020 Nov 23;11:5934. doi: 10.1038/s41467-020-19806-6 (PMC7683533; doi:10.1038/s41467-020-19806-6)
Supplement: Supplementary file 1 — Supplementary Information [file 41467_2020_19806_MOESM1_ESM.pdf]

Supplementary Information

# Curved neuromorphic image sensor array using a $MoS_2$-organic heterostructure inspired by the human visual recognition system

Changsoon Choi *et al.*

**The PDF file includes:**

Supplementary Notes 1-5, Supplementary Figures 1-23, Supplementary Tables 1-4, and Supplementary References.

# Supplementary Notes

## Supplementary Note 1. Optical analyses of the curved neuromorphic imaging device

The ray tracing simulation for the curved neuromorphic imaging device in comparison with the conventional imaging device was carried out by using commercial software (OpticStudio 16.0, Radiant ZEMAX LLC, USA). In the simulation, the operating wavelengths were set as 450 nm, 550 nm, and 650 nm for considering the polychromatic tracing simulation. The parallel rays were launched from infinite distances with different angles (*e.g.*, 0°, 7°, 14°, and 20°). The aperture size was set as F-number of 4. The detailed optical parameters for the simulation, such as radii, thicknesses, and materials, are summarized in Supplementary Tables 1 and 2.

In the conventional imaging device, the complicated multi-lens optics (*e.g.*, double Gauss lens) is required to reduce the spherical aberration, originated from the mismatch between the curved focal plane and the flat image sensor array[1]. For example, the double Gauss lens used in the conventional imaging devices is composed of seven lenses (Supplementary Fig. 3a), resulting in the high cost, large module size, and heavy weight as well as the complicated system construction. In contrast, the curved neuromorphic imaging device, inspired by the human vision, enables aberration-free imaging based on the single-lens optics[1] because the curved neuromorphic image sensor array (cNISA) matches with the curved focal plane of a single plano-convex lens (Supplementary Fig. 3b).

## Supplementary Note 2. Analytical model for the time-dependent photocurrent generation

The time-dependent photocurrent generation of pV3D3-PTr and $Al_2O_3$-PTr can be fitted

by an analytical model. The analytical model is given as:

$$I_{ph}(t) = I_1(1\text{-exp}(-t/\tau_1)) + I_2(1\text{-exp}(-t/\tau_2))$$

where $\tau_1$, $\tau_2$ are time constants, $I_1$, $I_2$ are coefficients of the photocurrent, and $t$ is the time for light irradiation[2].

Based on the photocurrent measurement data in Fig. 2f, the time constants ($\tau_1$, $\tau_2$) and the ratio of photocurrent coefficients ($I_2/I_1$) of pV3D3-PTr and $Al_2O_3$-PTr were estimated. The estimated $\tau_1$, $\tau_2$, and $I_2/I_1$ of pV3D3-PTr are 0.68 sec, 12.85 sec, and 11.03, respectively, and those of $Al_2O_3$-PTr are 0.80 sec, 4.39 sec, and 0.95, respectively.

Using these estimated parameters, we can analyze the contribution from each photocurrent term in the model to the overall photocurrent, *i.e.*, the relative contribution by $I_{ph,1}(t)$, *i.e.*, $I_1(1\text{-exp}(-t/\tau_1))$, and $I_{ph,2}(t)$, *i.e.*, $I_2(1\text{-exp}(-t/\tau_2))$, to $I_{ph}(t)$. To graphically analyze the contribution of each term, we co-plotted both individual photocurrent terms with the overall photocurrent. Photocurrents of pV3D3-PTr are plotted in Supplementary Fig. 7a, and those of $Al_2O_3$-PTr are plotted in Supplementary Fig. 7b.

$I_{ph,1}(t)$ increases rapidly but becomes saturated shortly (blue dotted line in Supplementary Figs. 7a and 7b). In contrast, $I_{ph,2}(t)$ increases quasi-linearly without saturation (red dotted line in Supplementary Figs. 7a and 7b). The shape of the overall photocurrent is determined by the relative contribution of each term. For example, if the contribution of $I_{ph,2}(t)$ is dominant, the overall photocurrent increases quasi-linearly. If not, the overall photocurrent increases nonlinearly and is saturated shortly.

In case of pV3D3-PTr, $I_{2,pV3D3}$ is an order of magnitude larger than $I_{1,pV3D3}$ ($I_{2,pV3D3}/I_{1,pV3D3}$ ~ 11.03). In addition, $\tau_{2,pV3D3}$ is 18.90 times larger than $\tau_{1,pV3D3}$. As a result, $I_{ph,2}(t)$ is much larger than $I_{ph,1}(t)$, which leads to the quasi-linear increase of the overall photocurrent (Supplementary Fig. 7a). And, the overall photocurrent can be approximated as $I_{ph}(t) \cong$

$I_{2,pV3D3}(t/\tau_{2,pV3D3})$ for small $t$ (*i.e.*, $t/\tau_{2,pV3D3} \ll 1$).

However, the photocurrent increase of $Al_2O_3$-PTr is different from that of pV3D3-PTr. First, $I_{2,Al2O3}$ and $I_{1,Al2O3}$ are comparable ($I_{2,Al2O3}/I_{1,Al2O3} \sim 0.95$). Furthermore, $\tau_{2,Al2O3}$ (4.39 sec) is smaller than $\tau_{2,pV3D3}$ (12.85 sec). Therefore, the photocurrent of $Al_2O_3$-PTr increases nonlinearly as shown in Supplementary Fig. 7b.

**Supplementary Note 3. Computational analyses of the $MoS_2$-pV3D3 heterostructure and the $MoS_2$-$Al_2O_3$ heterostructure**

We performed *ab initio* density functional theory (DFT) calculations using the Vienna *Ab initio* simulation package (VASP) to compute the electronic structure of the $MoS_2$-pV3D3 heterostructure and the $MoS_2$-$Al_2O_3$ heterostructure[3,4]. The pV3D3 chain, which consists of three units of V3D3, is placed on top of $MoS_2$ surface in a supercell with a size of 6 × 6 × 1 (Fig. 2k). The $Al_2O_3$, which consists of seven layers of Al atoms in the aperiodic direction connected through O atoms, is placed in a supercell with a size of 3 × 3 ×1 of the $MoS_2$ unit cell (Supplementary Fig. 13b). The Perdew−Burke−Ernzerhof (PBE) exchange-correlation functional was used[4]. The projected augmented wave (PAW) pseudopotentials were used with an energy cutoff of 450 eV. Both heterostructures were fully relaxed until the force on each atom reached 0.05 eV $Å^{-1}$, and the energy is converged to $10^{-6}$ eV by using Gamma-point-centered k-point of 2 × 2 × 1. In all calculations, the vacuum level was kept at least 18 Å to suppress unphysical interactions in the aperiodic direction. We used DFT-D3 to apply Gamma-point-centered k-point of 4 × 4 × 1 to the fully relaxed structures for capturing dispersion interactions and computing the density of states and charge differences[5]. The band alignment was computationally obtained based on the reference of the vacuum energy and the work

function of each material. Then, the band energy levels are aligned with respect to the vacuum energy. The charge differences were visualized by using the VESTA package[6].

To determine the exciton binding energy, we performed the many-body GW+BSE (Bethe-Salpeter Equation) calculations using the BerkeleyGW package[7]. The exciton binding energy was defined as the difference between the electronic bandgap energy and the first excitation energy of the optical bandgap. The mean field simulations were initiated by using the Quantum Espresso[8] package based on DFT at the PBE level. We used optimized norm-conserving pseudopotentials[9] to construct wavefunctions with a plane-wave kinetic energy cutoff of 60 Ry and a Gamma-point-centered k-point grid of 4 × 4 × 1. The dielectric cutoff was set to 7 Ry.

In the computational analyses, the exciton binding energies of the $MoS_2$-pV3D3 heterostructure and the $MoS_2$-$Al_2O_3$ heterostructure were calculated to be 0.43 eV and 0.24 eV, respectively. Such large exciton binding energies could lead to efficient charge transfer at the interface caused by the real-space localization of the electron-hole pairs because the electron-hole pair would find lower energy sites to accommodate the large binding energy, although the efficient charge separation processes overcoming the large exciton binding energy in 2D materials have not been fully understood[10-12]. In addition, the $MoS_2$-pVD3 heterostructure exhibits type-II band alignment, of which the valence band of pV3D3 is lower than that of $MoS_2$. Therefore, the charge separation at the interface of $MoS_2$-pV3D3 heterostructure is probable (Fig. 2j). In contrast, the charge separation at the interface of $MoS_2$-$Al_2O_3$ heterostructure is less probable (Supplementary Fig. 13a) because of its type-I band alignment, of which the valence band of $Al_2O_3$ is higher than that of $MoS_2$.

The interfacial charge density, $\Delta\rho = \rho_{MoS2,B} - \rho_{MoS2} - \rho_B$ where the subscript B is either dielectric (*e.g.*, pV3D3 or $Al_2O_3$), was also computed to investigate the charge density distribution and potential charge trapping sites at the interface of $MoS_2$-pV3D3 heterostructure

and $MoS_2$-$Al_2O_3$ heterostructure. The averaged interfacial charge density in planes normal to the interface was calculated to quantitatively analyze the charge density in the vertical direction. The plane-averaged interfacial charge density distribution in the $MoS_2$-pV3D3 heterostructure shows that the high-density electrons are localized at $MoS_2$ side and the high-density holes are localized at pV3D3 side. On the other hand, in the $MoS_2$-$Al_2O_3$ heterostructure, electronic localization is observed at the interface between $MoS_2$ and $Al_2O_3$, *i.e.*, fluctuation of electron and hole peaks within the interface.

**Supplementary Note 4. Mechanical analyses of the curved neuromorphic image sensor array**

Finite element analysis for the strain distribution of cNISA on a hemispherical substrate was carried out by using COMSOL Multiphysics software (COMSOL Inc., USA). The mesh-patterned PI film whose thickness is 2 μm was deformed along the hemispherical substrate whose bending radius is 11.3 mm. It was assumed that the conformal contact was made. First principle mechanical strain with plasticity of PI was calculated by using an initial yield stress of 24.8 MPa, an isotropic tangent modulus of 1.39 GPa, and a Poisson's ratio of 0.4.

**Supplementary Note 5. Experimental characterizations of the $MoS_2$-pV3D3 heterostructure and the $MoS_2$-$Al_2O_3$ heterostructure**

The synthesized $MoS_2$ was characterized by the Raman and photoluminescence (PL) spectroscopy. The Raman spectrum of as-grown $MoS_2$ (black line in Supplementary Fig. 21) clearly shows two distinctive Raman peaks, *e.g.,* S-Mo-S in-plane mode (~386 $cm^{-1}$; $E^{1}_{2g}$) and out-of-plane mode (~406 $cm^{-1}$; $A_{1g}$). In Raman spectra of the $MoS_2$-pV3D3 heterostructure and the $MoS_2$-$Al_2O_3$ heterostructure (red line and blue line in Supplementary Fig. 21, respectively),

$E^{1}_{2g}$ was barely shifted by pV3D3 and $Al_2O_3$ deposition, implying the residual strain caused by the deposition process is small[13]. However, $A_{1g}$ was red-shifted in both spectra, implying the deposited dielectric layers induced a slight doping effect to the $MoS_2$ layer[14,15].

The PL spectrum of as-grown $MoS_2$ exhibits a dominant peak at ~1.87 eV that corresponds to the optical bandgap energy of monolayer $MoS_2$ (black line in Supplementary Fig. 22a). PL spectrum of the $MoS_2$-$Al_2O_3$ heterostructure exhibits the reduced intensity (blue line in Supplementary Fig. 22a), implying the PL quenching effect by $Al_2O_3$ layer[16]. On the other hand, PL spectrum of the $MoS_2$-pV3D3 heterostructure exhibits a red-shifted peak with a wider full-width half-maximum (FWHM) (red line in Supplementary Fig. 22a). We analyzed PL spectra of the $MoS_2$-pV3D3 heterostructure in comparison to that of as-grown $MoS_2$ (Supplementary Figs. 22b and 22c), and A exciton (A) and charged exciton ($A^-$; trion) peaks are extracted by Gaussian fitting. Although PL intensity of the $MoS_2$-pV3D3 heterostructure is consistent with that of the as-grown $MoS_2$, the intensity ratio between charged exciton and A exciton ($I_{A^-}/I_A$) increases by the pV3D3 deposition, which suggests small amount of electron doping effect by the deposited pV3D3 layer[13].

# Supplementary Figures

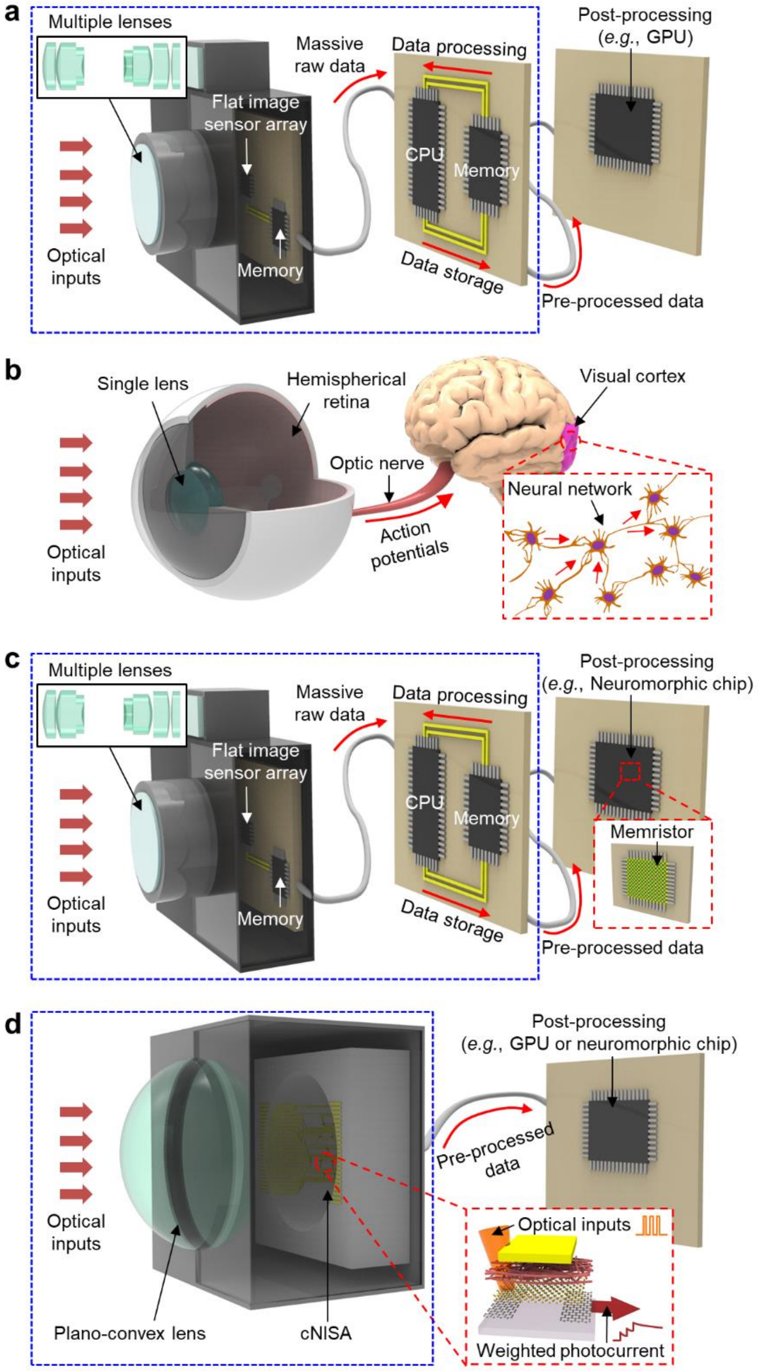


**Supplementary Figure 1 | Imaging and data processing systems.** Schematic illustration of the imaging and data processing systems. **a**, Conventional imaging and data processing system, comprised of multiple lenses, an image sensor array, memory modules, and processing units (*e.g.*, GPU). **b**, Human visual recognition system, comprised of a single human-eye lens, a hemispherical retina, optic nerves, and a neural network in the visual cortex. **c**, Recently-

proposed imaging and data processing system, comprised of multiple lenses, an image sensor array, memory modules, and processing units (*e.g.*, neuromorphic chip). **d**, Curved neuromorphic imaging device, comprised of a single plano-convex lens, cNISA, and a post-processor.

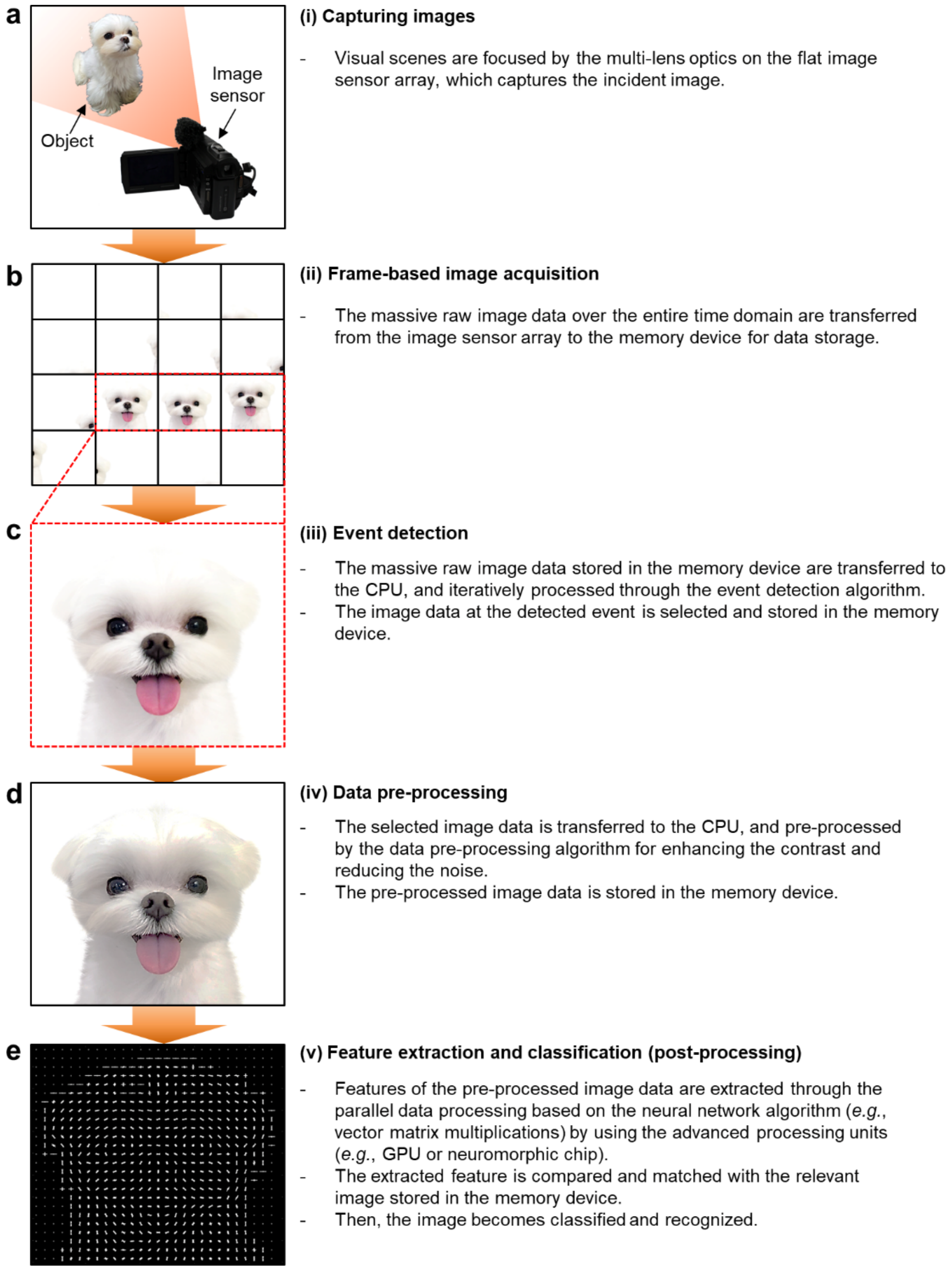


**Supplementary Figure 2 | Image recognition process in conventional image sensors and data processing devices.** Imaging and recognition steps, including image acquisition, event detection, data pre-processing, and data post-processing, in the conventional image sensors and data processing devices.

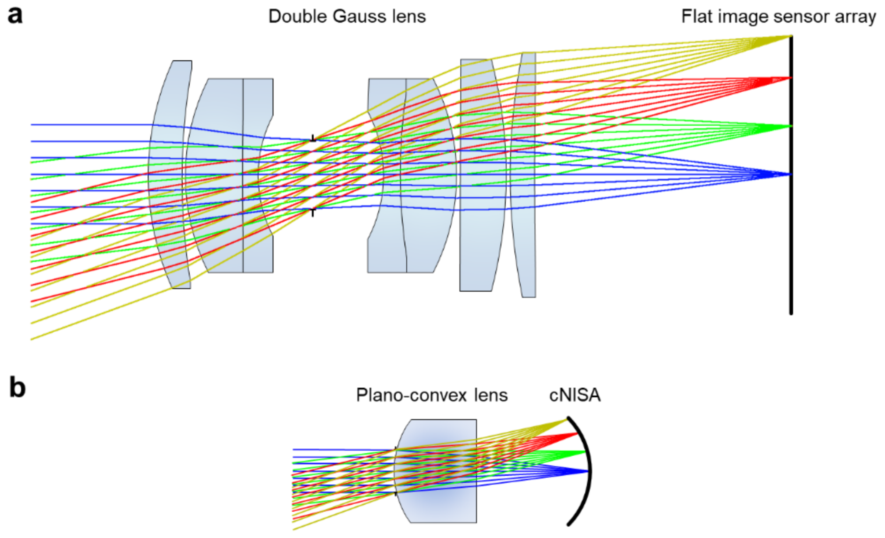


**Supplementary Figure 3 | Ray tracing simulation. a**, Ray tracing simulation for the complicated optics in the conventional imaging device. **b**, Ray tracing simulation for the simplified optics in the curved neuromorphic imaging device.

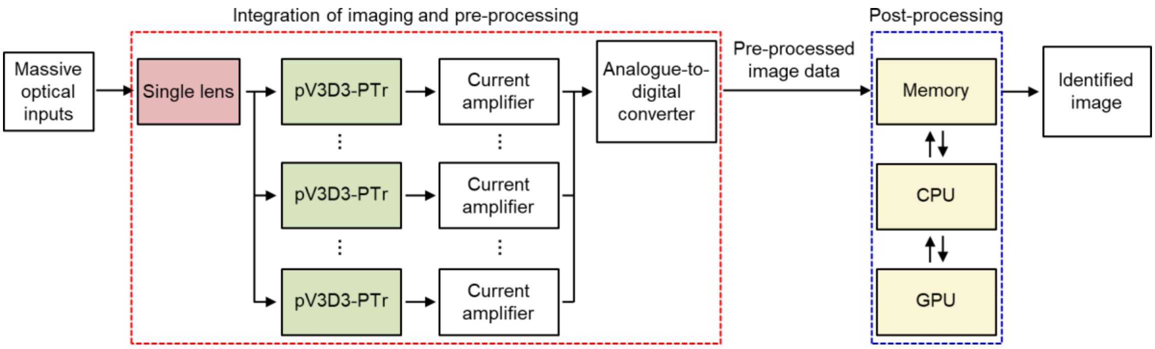


**Supplementary Figure 4 | Overall architecture for imaging and recognition.** Schematic diagram that describes the overall architecture for image acquisition, data pre-processing, and data post-processing by using cNISA, a customized data acquisition system, and post-processors.

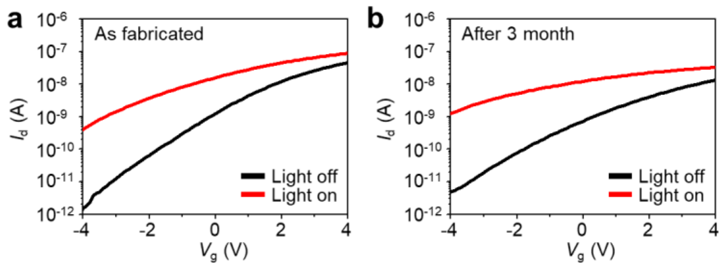


**Supplementary Figure 5 | Time-dependent electrical characterization of pV3D3-PTr. a**, Transfer curves of as-fabricated pV3D3-PTr under the light-off and light-on condition. **b**, Transfer curves of pV3D3-PTr stored in the ambient condition for three months. Its transfer characteristics are consistent with the original transfer curves.

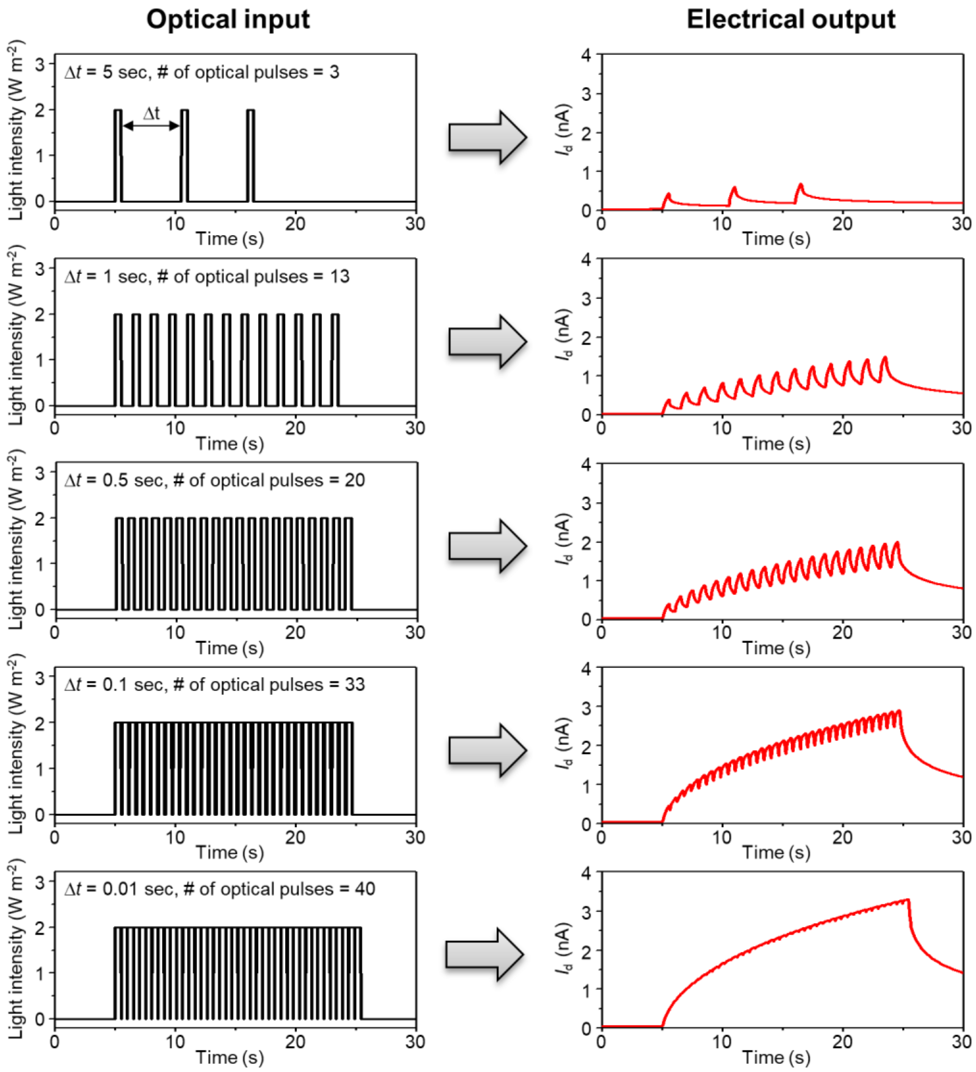


**Supplementary Figure 6 | Photocurrent generation and decaying characteristics of pV3D3-PTr for the optical inputs of various frequencies.** Photocurrent generation and decaying characteristics of pV3D3-PTr in response to a series of optical inputs with various time intervals.

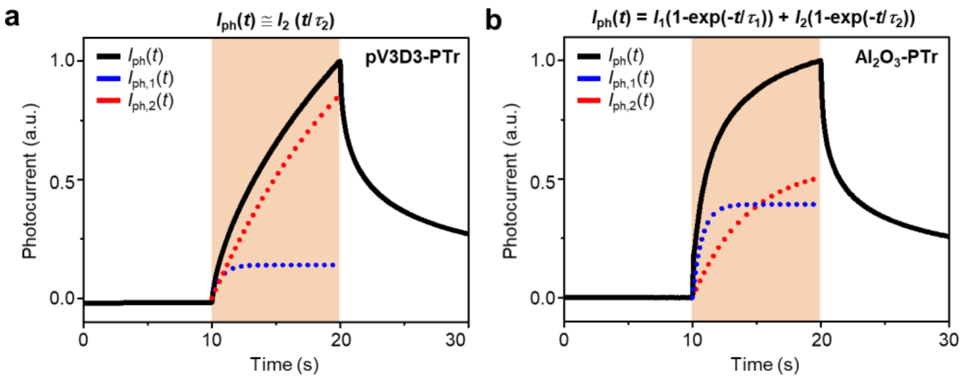


**Supplementary Figure 7 | Modeling of the time-dependent photocurrent generation. a**,**b**, Photocurrent generation characteristics of pV3D3-PTr (**a**) and $Al_2O_3$-PTr (**b**). The black line shows the overall photocurrent, and the blue and red dotted lines show the individual photocurrent terms in the analytical model. (*i.e.*, $I_{ph}(t) = I_{ph,1}(t) + I_{ph,2}(t)$ in which $I_{ph,1}(t) = I_1(1 - \exp(-t/\tau_1))$ and $I_{ph,2}(t) = I_2(1 - \exp(-t/\tau_2))$).

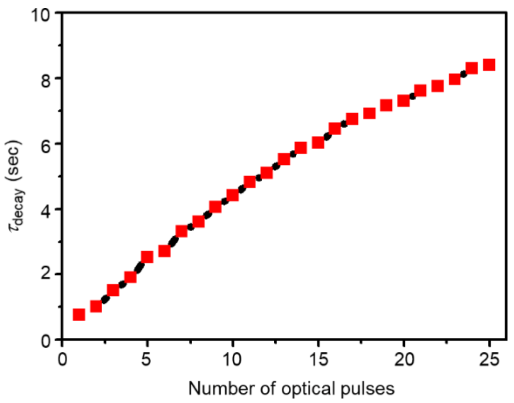


**Supplementary Figure 8 | Prolonged photocurrent decay with more optical inputs.** The decay time constant ($\tau_{decay}$) of pV3D3-PTr increases as the number of applied optical pulses increases.

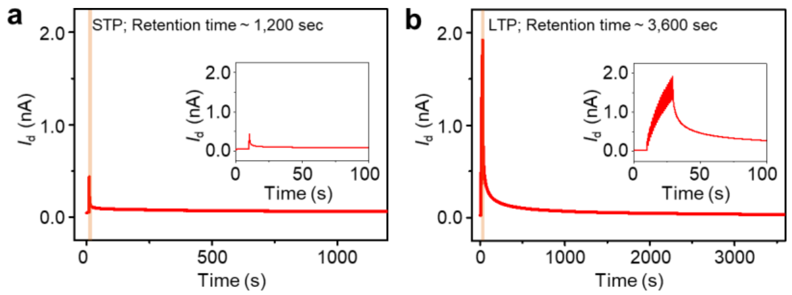


**Supplementary Figure 9 | Retention characteristics. a**, Photocurrent decay characteristics of pV3D3-PTr in response to a single optical pulse with 0.5 sec duration. **b**, Photocurrent decay characteristic of pV3D3-PTr in response to 20 optical pulses with 0.5 sec durations and 0.5 sec intervals.

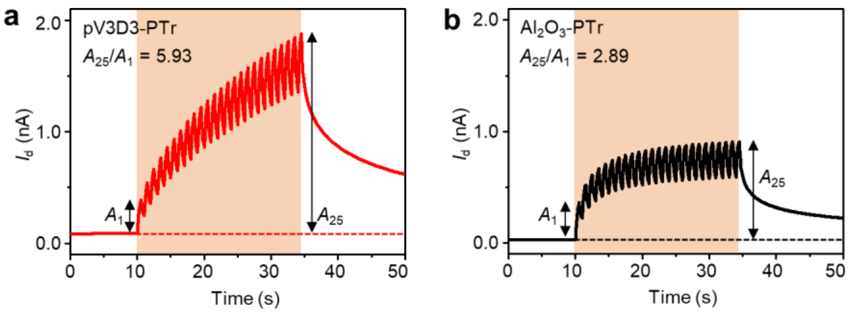


**Supplementary Figure 10 | Synaptic weight of pV3D3-PTr and $Al_2O_3$-PTr under 25 optical pulses. a**,**b**, Photocurrent generation and decaying characteristics of pV3D3-PTr (**a**) and $Al_2O_3$-PTr (**b**) upon the irradiation of 25 optical pulses. Synaptic weights can be calculated as $A_{25}/A_1$.

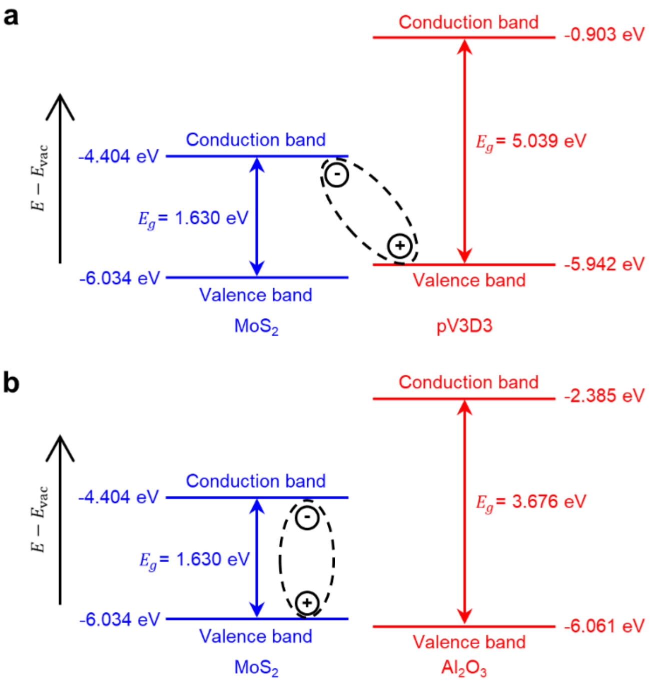


**Supplementary Figure 11 | Band structure of the $MoS_2$-pV3D3 heterostructure and the $MoS_2$-$Al_2O_3$ heterostructure. a**,**b**, Computationally obtained electronic band structure of the $MoS_2$-pV3D3 heterostructure (**a**) and the $MoS_2$-$Al_2O_3$ heterostructures (**b**). The $MoS_2$-pV3D3 heterostructure exhibits type-II band alignment, while the $MoS_2$-$Al_2O_3$ heterostructure exhibits type-I band alignment.

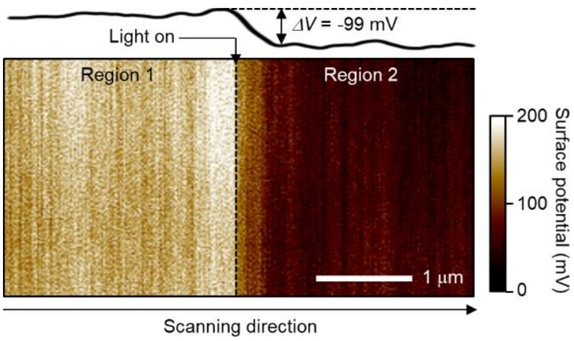


**Supplementary Figure 12 | Kelvin probe force microscopy measurement.** Surface potential measurement of the $MoS_2$-pV3D3 heterostructure. The light condition was changed from off-condition (region 1) to on-condition (region 2) during the scanning (*i.e.*, light was turned on during the measurement). The black arrow at the bottom indicates the scanning direction.

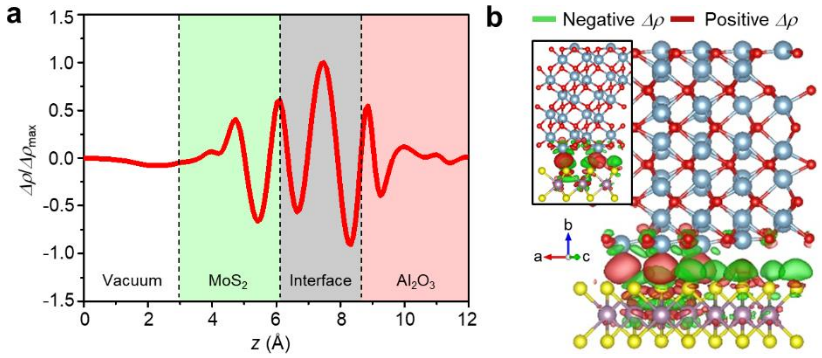


**Supplementary Figure 13 | Computed interfacial charge density of the $MoS_2$-$Al_2O_3$ heterostructure.** **a**, Computed interfacial charge density in the $MoS_2$-$Al_2O_3$ heterostructure (*i.e.*, $\Delta\rho$) depending on the distance in the aperiodic lattice direction. The charge density is computed using the DFT-D3 method. **b**, Contour plots of the charge density difference in planes normal to the interface in the $MoS_2$-$Al_2O_3$ heterostructure. The green and red contours imply potential hole trapping and electron trapping sites, respectively. The inset shows a side view of Supplementary Fig. 13b.

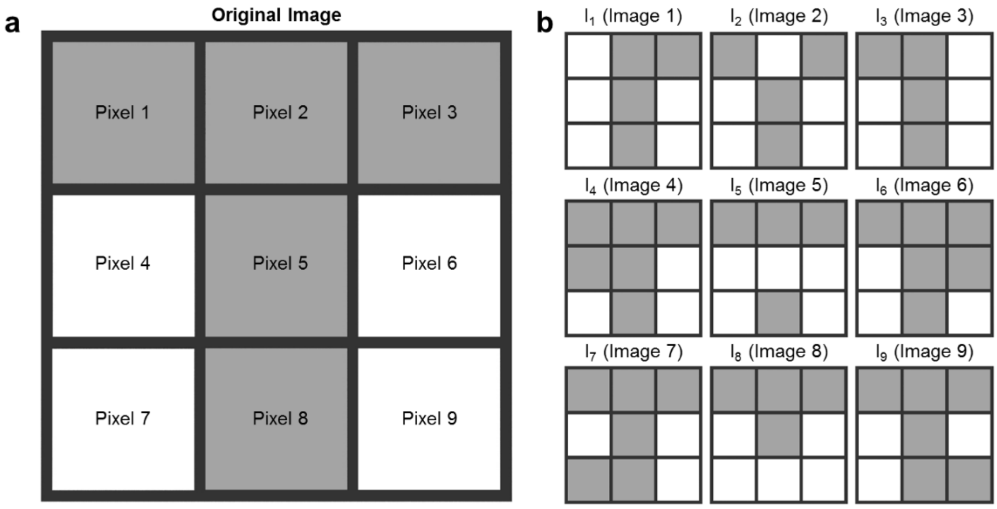


**Supplementary Figure 14 | A set of noisy optical inputs for image acquisition and neuromorphic data pre-processing. a**, Original image whose shape is the character 'T'. **b**, A set of noisy optical inputs (*i.e.*, T-shaped images with a flipped pixel).

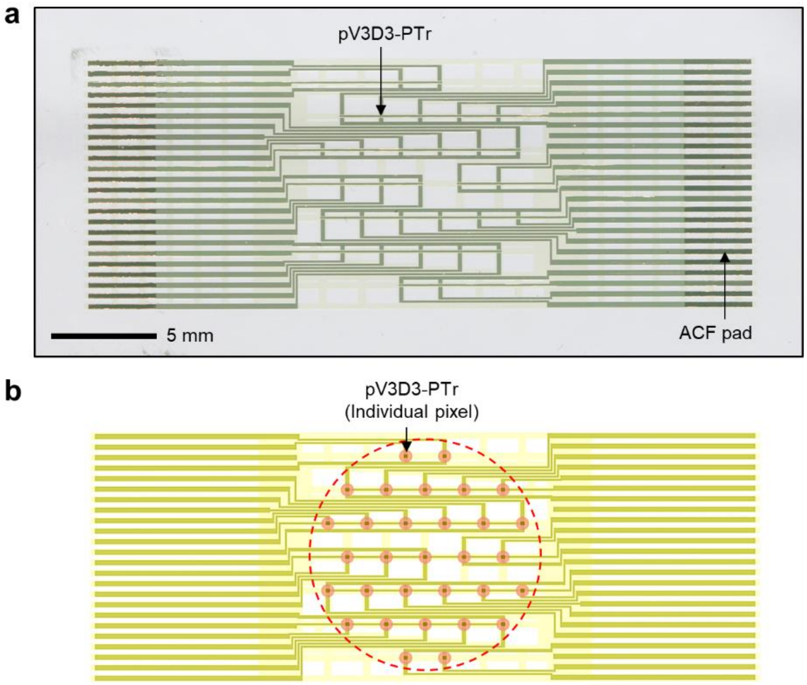


**Supplementary Figure 15 | Neuromorphic image sensor array. a**, Optical camera image of the fabricated array that consists of 31 pixels of pV3D3-PTrs. **b**, Schematic illustration of the 31 pixels of pV3D3-PTrs, which shows the circular pixel distribution in the array.

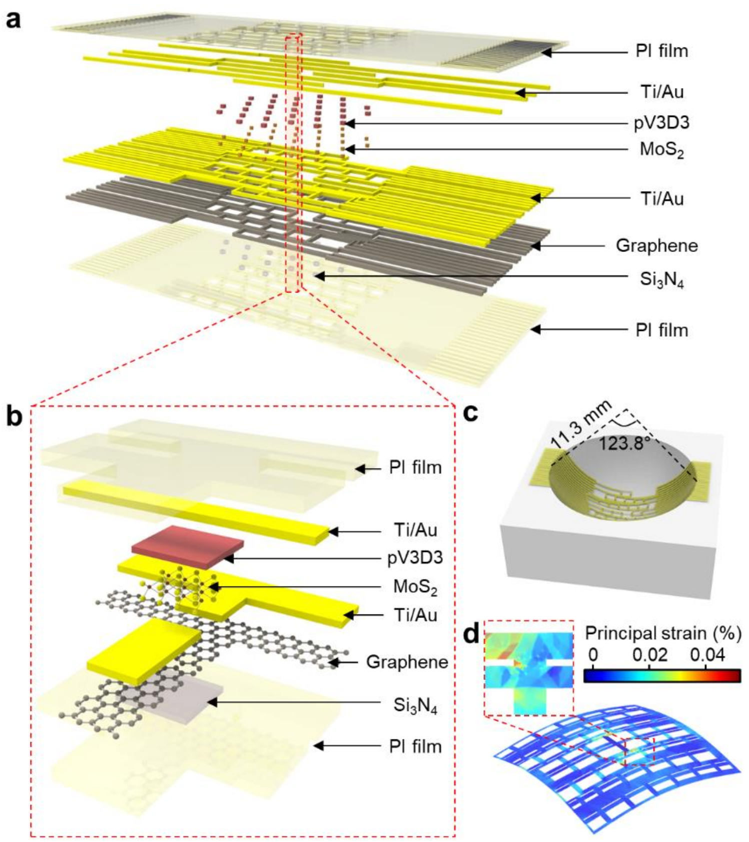


**Supplementary Figure 16 | Device design. a**, Schematic illustration showing the design of the pV3D3-PTr arrays. **b**, Schematic illustration showing an individual pixel of cNISA. **c**, Schematic illustration of cNISA on the hemispherical substrate. Its radius of curvature is 11.3 mm, and its subtended angle is 123.8°. **d**, First principal strain distribution calculation. The inset shows the first principal strain distribution in the individual pixel of cNISA.

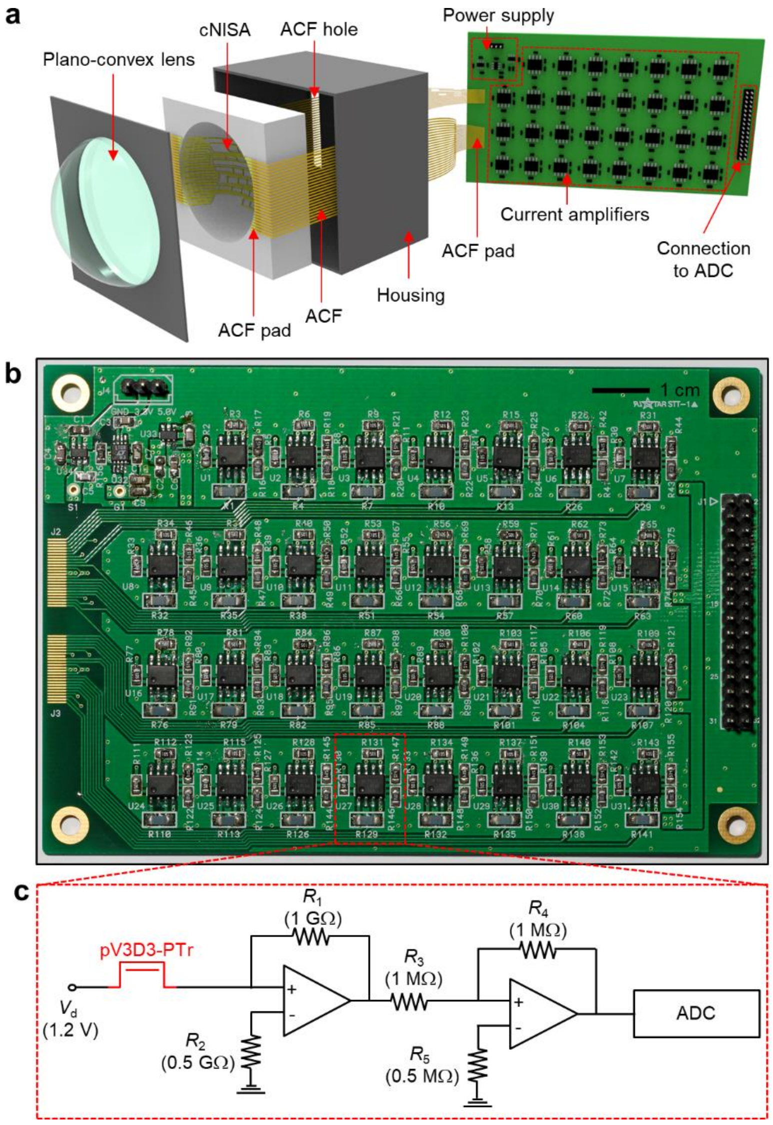


**Supplementary Figure 17 | Customized data acquisition system. a**, Exploded schematic illustration that shows the electrical connection between cNISA and the customized data acquisition system through the ACF. **b**, Optical camera image of the customized data acquisition system, in which current amplifiers and power supplying chips are assembled on PCB. **c**, Schematic diagram of the current amplifier used in the customized data acquisition system for translating the photocurrent into amplified voltage signals.

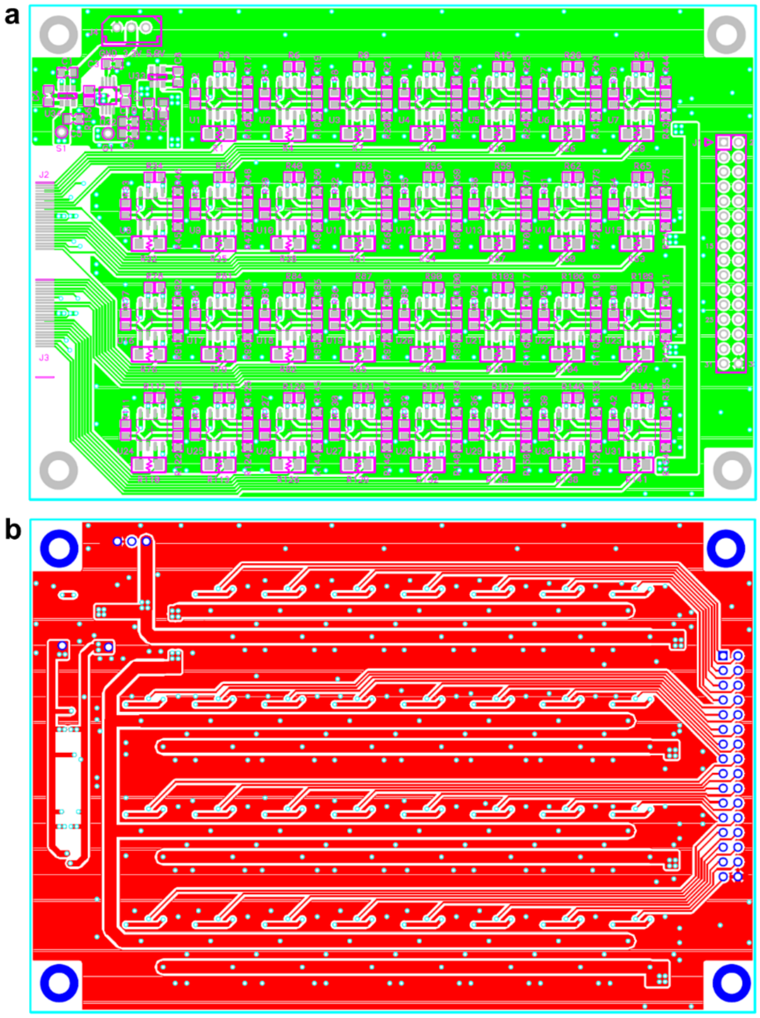

**Supplementary Figure 18 | Layout of a customized data acquisition system. a**,**b**, Layout of the front (**a**) and back (**b**) of the customized data acquisition system. U1-U31 indicate the current amplifiers (*i.e.*, transimpedance amplifiers and inverters), U32-U34 indicate the voltage regulators, and J1-J3 indicate the connection to external devices.

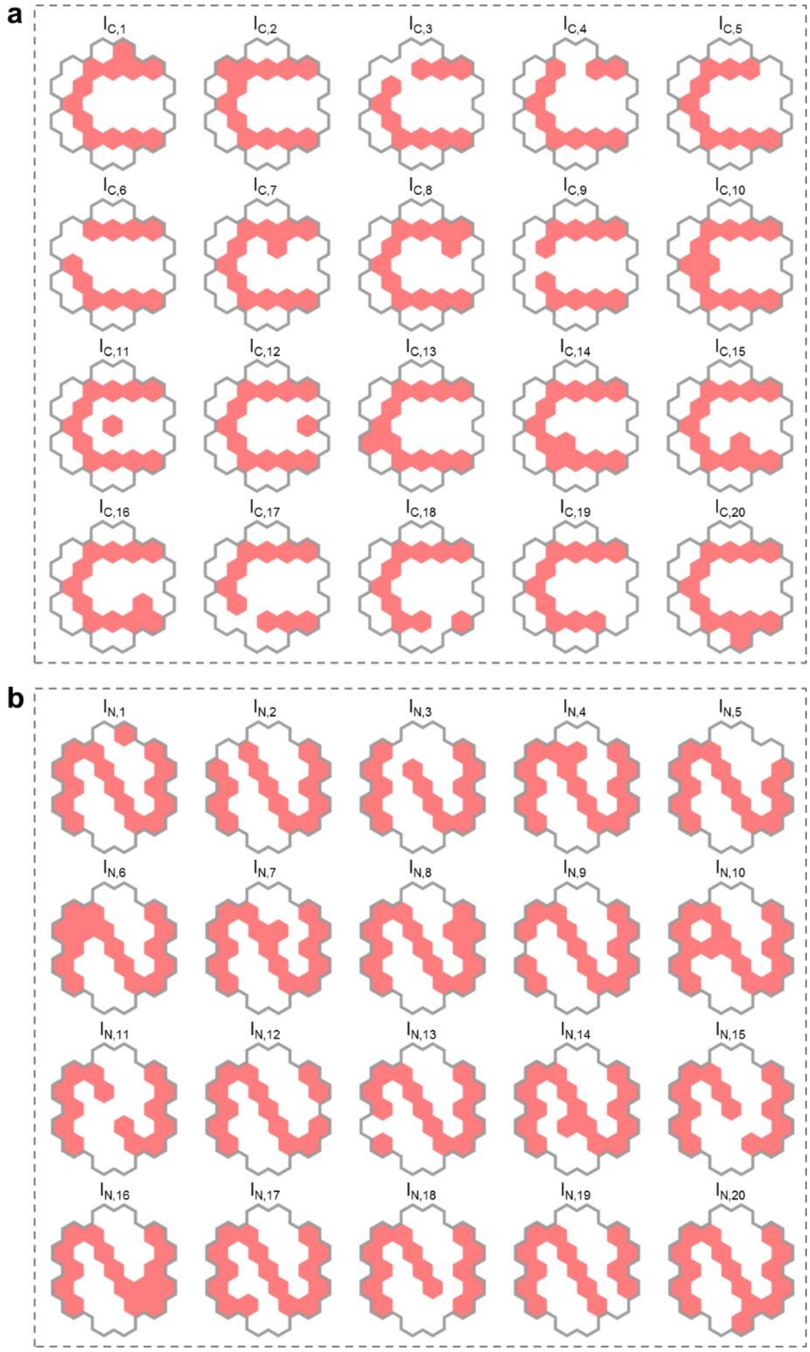


**Supplementary Figure 19 | A set of noisy optical inputs for neuromorphic imaging demonstrations. a**,**b**, Schematic illustrations of a set of noisy optical inputs (*e.g.*, C-shaped noisy optical inputs (**a**) and N-shaped noisy optical inputs (**b**)).

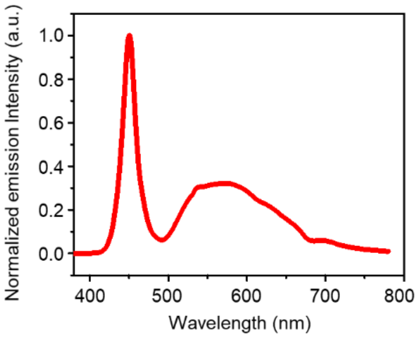


**Supplementary Figure 20 | Characterization of a white light-emitting diode.** Normalized emission spectrum of the white light-emitting diode which was used as a light source.

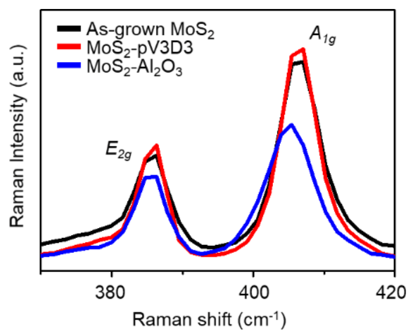


**Supplementary Figure 21 | Raman spectroscopy.** Raman spectroscopy of as-grown $MoS_2$, the $MoS_2$-pV3D3 heterostructure, and the $MoS_2$-$Al_2O_3$ heterostructure.

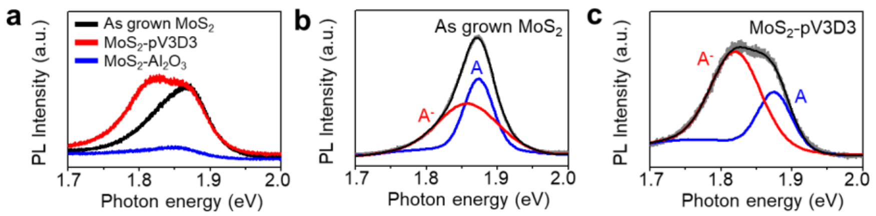


**Supplementary Figure 22 | Photoluminescence analysis.** **a**, PL spectra of as-grown $MoS_2$, the $MoS_2$-pV3D3 heterostructure, and the $MoS_2$-$Al_2O_3$ heterostructure. **b**,**c**, PL spectrum of as-grown $MoS_2$ (**b**) and $MoS_2$ on pV3D3 (**c**). The gray line shows the experimental data. The red and blue lines correspond to the fitted peaks by Gaussian functions. The black line shows the summation of the fitted peaks.

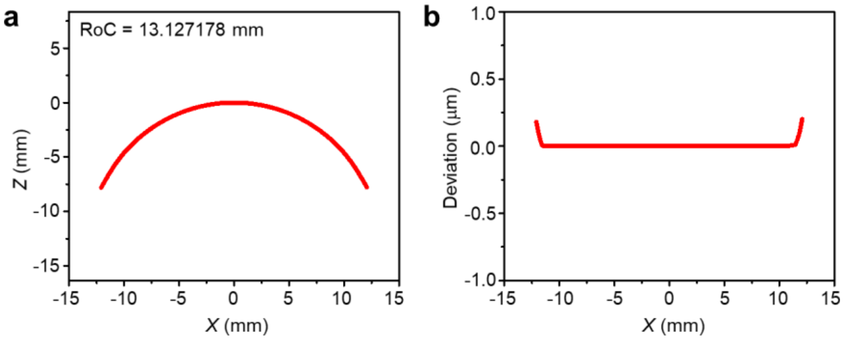


**Supplementary Figure 23 | Surface profile of the plano-convex lens. a**, Surface profile of the plano-convex lens. **b**, Surface roughness of the plano-convex lens. The best fit radius of curvature is 13.127178 mm, the RMS value of the roughness is 0.2798 μm, and the peak-to-peak variation of the roughness is 4.4593 μm.

# Supplementary Tables

| Table 1 \| Optics for curved neuromorphic imaging device | | | | |
|---|---|---|---|---|
| **Component** | **Radius (mm)** | **Thickness (mm)** | **Material** | **Semi-diameter (mm)** |
| Object | Infinity | Infinity | - | Infinity |
| Aperture | Infinity | 0.000 | - | 2.532 |
| 1 | 13.127 | 12.220 | N-BK7 | 8.000 |
| 2 | Infinity | 17.045 | - | 3.653 |
| Image sensor | -11.340 | - | - | 9.500 |

**Supplementary Table 1 | Lens information of the curved neuromorphic imaging device.** The radii, thicknesses, materials, and semi-diameters of the comprising lens component in the curved neuromorphic imaging device shown in Supplementary Fig. 3b.

| Table 2 \| Optics for conventional imaging device | | | | |
|---|---|---|---|---|
| **Component** | **Radius (mm)** | **Thickness (mm)** | **Material** | **Semi-diameter (mm)** |
| Object | Infinity | Infinity | - | Infinity |
| 1 | 34.333 | 4.435 | N-BASF2 | 14.655 |
| 2 | 78.925 | 0.381 | - | 13.700 |
| 3 | 27.554 | 7.452 | N-LAK8 | 12.424 |
| 4 | 592.999 | 2.032 | SF2 | 9.879 |
| 5 | 16.807 | 7.036 | - | 7.669 |
| Aperture | Infinity | 9.276 | - | 4.465 |
| 7 | -16.965 | 2.032 | SF2 | 8.167 |
| 8 | 69.433 | 7.394 | N-LAK33 | 10.825 |
| 9 | -25.644 | 0.381 | - | 12.427 |
| 10 | Infinity | 5.989 | N-LAK33 | 13.962 |
| 11 | -58.641 | 0.381 | - | 14.888 |
| 12 | 79.263 | 3.348 | N-LAK8 | 15.667 |
| 13 | 699.404 | 33.035 | - | 15.720 |
| Image sensor | Infinity | - | - | 30.309 |

**Supplementary Table 2 | Lens information of the conventional imaging device.** The radii, thicknesses, materials, and semi-diameters of the comprising lens components in the conventional imaging device shown in Supplementary Fig. 3a.

| Table 3 \| Specifications of neuromorphic image sensors | | | | | |
|---|---|---|---|---|---|
| **Image sensor** | **Neuromorphic image sensor from Ref. 1** | **Neuromorphic image sensor from Ref. 14** | **Neuromorphic image sensor from Ref. 24** | **Neuromorphic image sensor from Ref. 36** | **Curved neuromorphic imaging device** |
| Device type | Optoelectronic memory | Phototransistor | Photodiode | Phototransistor | Phototransistor |
| Materials | $MoO_x$ | Amorphous IGZO | $WSe_2$ | $MoS_2$ | $MoS_2$ |
| Format | Array | Single device | Array | Array | Array |
| Optical inputs | UV | UV | Visible laser | Visible spectrum | Visible spectrum |
| Number of pixels | 8×8 | 1 | 3×3 | 32×32 | 31 |
| Optics | N.A. | N.A. | N.A. | N.A. | Single-lens optics (Plano-convex lens) |
| Applications | Image acquisition, pre-processing | N.A. | Pattern classification, autoencoding | Image acquisition, post-processing | Image acquisition, pre-processing |
| Reference | [1] | [14] | [24] | [36] | This work |

**Supplementary Table 3 | Specifications of neuromorphic image sensors.** Comparison of the current curved neuromorphic imaging device with the state-of-the-art neuromorphic image sensors in terms of the device type, key materials, the device format, the type of optical inputs, the number of pixels, information for optics, and information for their applications.

| Table 4 \| Components in customized data acquisition system | | | |
|---|---|---|---|
| **Component** | **Type** | **Value** | **Model** |
| U1-U31 | Current amplifier (Transimpedance amplifier, Inverter) | N.A. | LMC662 |
| U32 | Voltage regulator | -2.0 V | LTC1550 |
| U33 | Voltage regulator | -5.0 V | LTC1983 |
| U34 | Voltage regulator | 1.2 V | LTC3250 |
| J1 | Connection to ADC | N.A. | N.A. |
| J2, J3 | ACF pad | N.A. | N.A. |

**Supplementary Table 4 | Components used in a customized data acquisition system.** Detailed information of the components used in the customized data acquisition system shown in Supplementary Fig. 18.

## Supplementary References


1. Choi, C. *et al.* Human eye-inspired soft optoelectronic device using high-density $MoS_2$-graphene curved image sensor array. *Nat. Commun.* **8**, 1664 (2017).
2. Amit, I. *et al.* Role of charge traps in the performance of atomically thin transistors. *Adv. Mater.* **29**, 1605598 (2017).
3. Kresse, G. & Furthmüller, J. Efficient iterative schemes for *ab initio* total-energy calculations using a plane-wave basis set. *Phys. Rev. B* **54**, 11169-11186 (1996).
4. Gaspar, R. & Nagy, A. Local-density-functional approximation for exchange-correlation potential. *Theoretica chimica acta.* **72**, 393-401 (1987).
5. Grimme S., Antony J., Ehrlich S. & Krieg H. A consistent and accurate *ab initio* parametrization of density functional dispersion correction (DFT-D) for the 94 elements H-Pu. *J. Chem. Phys.* **132**, 154104 (2010).
6. Momma K. & Izumi F., VESTA: a three-dimensional visualization system for electronic and structural analysis. *J. Appl. Cryst.* **41**, 653-658 (2008).
7. Deslippe, J., Samsonidze, G., Strubbe, D. A., Jain, M., Cohen, M. L. & Louie, S. G. BerkeleyGW: A massively parallel computer package for the calculation of the quasiparticle and optical properties of materials and nanostructures. *Comput. Phys. Commun.* **183**, 1269-1289 (2012).
8. Giannozzi, P. *et al.* QUANTUM ESPRESSO: a modular and open-source software project for quantum simulations of materials. *J. Phys. Condens.* **21**, 395502 (2009).
9. Hamann, D. R. Optimized norm-conserving Vanderbilt pseudopotentials. *Phys. Rev. B* **88**, 085117 (2013).
10. Massicotte, M. *et al.* Dissociation of two-dimensional excitons in monolayer $WSe_2$. *Nat.*

*Commun.* **9**, 1633 (2018).

11. Zhu, X. *et al.* Charge transfer excitons at van der Waals interfaces. *J. Am. Chem. Soc.* **137**, 8313-8320 (2015).
12. Zhu, T. *et al.* Highly mobile charge-transfer excitons in two-dimensional $WS_2$/tetracene heterostructures. *Sci. Adv.* **12**, eaao3104 (2018).
13. Lin, Y. *et al.* Dielectric screening of excitons and trions in single-layer $MoS_2$. *Nano Lett.* **14**, 5569-5576 (2014).
14. Kukuscka, G. & Koltai, J. Theoretical investigation of strain and doping on the Raman spectra of monolayer $MoS_2$. *Phys. Status Solidi B* **254**, 1700184 (2017).
15. Cheng, L. *et al.* Sub-10nm tunable hybrid dielectric engineering on $MoS_2$ for two-dimensional material-based devices. *ACS Nano* **11**, 10243-10252 (2017).
16. Kim, S. Y., Yang, H. I. & Choi, W. Photoluminescence quenching in monolayer transition metal dichalcogenides by $Al_2O_3$ encapsulation. *Appl. Phys. Lett.* **113**, 133104 (2018).
